# Integrated genome based evaluation of safety and probiotic characteristics of *Lactiplantibacillus plantarum* YW11 isolated from Tibetan kefir

**DOI:** 10.3389/fmicb.2023.1157615

**Published:** 2023-04-20

**Authors:** Tariq Aziz, Muhammad Naveed, Khizra Jabeen, Muhammad Aqib Shabbir, Abid Sarwar, Yang Zhennai, Metab Alharbi, Abdulrahman Alshammari, Abdullah F. Alasmari

**Affiliations:** ^1^Beijing Advanced Innovation Center for Food Nutrition and Human Health, Beijing Engineering and Technology Research Center of Food Additives, Beijing Technology and Business University, Beijing, China; ^2^Department of Agriculture, University of Ioannina, Ioannina, Greece; ^3^Department of Biotechnology, Faculty of Science and Technology, University of Central Punjab, Lahore, Pakistan; ^4^Department of Pharmacology and Toxicology, College of Pharmacy, King Saud University, Riyadh, Saudi Arabia

**Keywords:** kefir, comparative genomics, cefoxitin, carbohydrate metabolism, genetics and genomics, pathogenic

## Abstract

The comparative genomic analysis of *Lactiplantibacillus plantarum* YW11 (*L. plantarum* YW11) isolated from Tibetan kefir involves comparison of the complete genome sequences of the isolated strain with other closely related *L. plantarum* strains. This type of analysis can be used to identify the genetic diversity among strains and to explore the genetic characteristics of the YW11 strain. The genome of *L. plantarum* YW11 was found to be composed of a circular single chromosome of 4,597,470 bp with a G + C content of 43.2%. A total of 4,278 open reading frames (ORFs) were identified in the genome and the coding density was found to be 87.8%. A comparative genomic analysis was conducted using two other *L. plantarum* strains, *L. plantarum* C11 and *L. plantarum* LMG21703. Genomic comparison revealed that *L. plantarum* YW11 shared 72.7 and 75.2% of gene content with *L. plantarum* C11 and *L. plantarum* LMG21703, respectively. Most of the genes shared between the three *L. plantarum* strains were involved in carbohydrate metabolism, energy production and conversion, amino acid metabolism, and transcription. In this analysis, 10 previously sequenced entire genomes of the species were compared using an *in-silico* technique to discover genomic divergence in genes linked with carbohydrate intake and their potential adaptations to distinct human intestinal environments. The subspecies pan-genome was open, which correlated with its extraordinary capacity to colonize several environments. Phylogenetic analysis revealed that the novel genomes were homogenously grouped among subspecies of l *Lactiplantibacillus. L. plantarum* was resistant to cefoxitin, erythromycin, and metronidazole, inhibited pathogens including Listeria monocytogenes, Clostridium difficile, Vibrio cholera, and others, and had excellent aerotolerance, which is useful for industrial operations. The comparative genomic analysis of *L. plantarum* YW11 isolated from Tibetan kefir can provide insights into the genetic characteristics of the strain, which can be used to further understand its role in the production of kefir.

## 1. Introduction

Lactic acid bacteria (LAB) are a group of microorganisms that are found everywhere in nature. Since they possess probiotic and antimicrobial properties, various species of LAB are added to a wide variety of foods to provide consumers with the opportunity to reap the associated health benefits (Sarwar et al., 2018; Rodrigo-Torres et al., 2019; Brandt et al., 2020; Tenea and Ortega, 2021). In addition, LAB species help to the safety of food by preventing the spread of microbes that cause unwanted spoiling or that are harmful. These organisms are also commonly employed in the fermentation procedures that are used to produce food. Barrangou et al. (2011), Fernández et al. (2013), Swain et al. (2014), Yépez et al. (2017), Adesulu-Dahunsi et al. (2018), and Goel et al. (2020). Additionally, LAB species are used in the medical and pharmaceutical industries, as well as in healthcare. LAB have demonstrated a variety of health promoting properties which can be used against intestinal illness, including inflammatory bowel diseases (IBD), as a result of their demonstrated antibacterial, immune modulating, and ability to control gut flora activities and these have been confirmed by different researchers (Yonekura et al., 2009; Hojsak et al., 2010; Kwon et al., 2010; Joo et al., 2011; Zhang et al., 2018). This is because these bacterial strains can regulate gut flora and control the bacteria that live there. *Lactobacillus* species are generally regarded as safe (GRAS) (Sarwar et al., 2018) for their usage in human bodies as well as their use in the food sector for an extended period as starter cultures. Among the *lactobacillus* species, *lactiplantibacillus plantarum* (previously known as *lactobacillus plantarum*) is the species that has received the most attention from researchers. It is possible to obtain *lactiplantibacillus plantarum* from a variety of sources, such as plant matter, fermented foods (yoghurt, pickles, cheese), meat products, fruit juices, the gastrointestinal tract of both humans and animals, and wine. In addition to that, this species is very useful for the fermentation of a wide range of foods (Stefanovic et al., 2017; Zhou et al., 2021; Wang et al., 2022).

The probiotic qualities of these strains are primarily responsible for all these activities, and because to the health promoting features that these strains possess, they have garnered the interest of researchers from all over the world (Jeong et al., 2022). More than 90 percent of the market for probiotics throughout the world was held by human products in 2021. According to the findings of experts, the worldwide market for probiotics might be worth $3.5 billion by the year 2026 (Wang et al., 2021). *L. plantarum* is a bacterium that dwells in the gastrointestinal tract (Kleerebezem et al., 2003; Aziz et al., 2022). It may be found in nearly any kind of environment. There are approximately 400 different bacterial species that make up the human stomach related framework. Some of these bacteria include *L. acidophilus* (Hatami et al., 2022), *L. pentosus*, *L. brevis*, *L. lactis* (Ashaolu and Reale, 2020), *L. amylovorus*, *L. casei, L. bulgaricus* (Albayrak and Duran, 2021), *L. fermentum*, *L. plantarum* and *L. rhamnosus* produces extracellular, exopolysaccharides, bacteriocins and lipoteichoic acids (Gupta et al., 2021).

The growing number of *lactiplantibacillus* strain genome sequences has shown their genetic potential for probiotic characteristics and adaptation to varied environmental conditions and stressors. Our Tibetan kefir strain *Lactiplantibacillus plantarum* YW11 regulates modulatory effects on gut dysbacteriosis, improves immunological response, and reduces inflammatory bowel illness, according to our newest findings (IBD) (Jian et al., 2020; Zhang et al., 2020). In addition to that it was also evident from another study that the *L. plantarum* YW11 has good tolerance to acid and bile stress (Jian et al., 2017). Correspondingly, we have also found that *L. plantarum* YW11 may be employed as a functional agent in the production of fermented dairy products with better textural stability and bioactivities such as cholesterol reducing, antioxidant, and antibiofilm properties (Zhang et al., 2020, 2022). Similarly, this strain *L. plantarum* YW11 has the competency of biotransformation of linoleic acid (LA) into conjugated linoleic acid (CLA) (Aziz et al., 2020). Most study has focused on viable probiotic strain effects and mechanisms. Scientists are growing interested in employing probiotics as immunologically active, microbiologically non-viable medications. It may be more effective, viable, and safer for therapeutic probiotic usage due to safety problems with the active metabolic form favoring bacterial translocation. The risk favored active metabolic form may explain these advantages. However, its genetic base for probiotic properties and adaptability is still mostly recognized (Moradi et al., 2020; Teame et al., 2020; De Jesus et al., 2022). Genomic-level studies can provide insights into the primary genetic factors and molecular mechanisms associated with the probiotic characteristics of these microorganisms, such as gastrointestinal tract survival, pathogen inhibition, and immunoregulation GIT survival, pathogen inhibition, and immunoregulation (Ventura et al., 2012; Salvetti and O’Toole, 2018; Castro-López et al., 2021).

Pan-probiosis, which compares the genomes of numerous probiotic bacterial strains, employs comparative genomics as an additional tool. This research aims to identify the best probiotic bacteria strains. Pan-genomic derivatives are a technique for discovering genes linked with probiotic properties that are either conserved across all strains of a certain bacteria or unique to a given genus or species (Rodenes et al., 2022). All known bacterial strains either have these genes, or all but one of them do not. Through the integration of phylogenomic research, studies can link genotypes and phenotypes to specific strains, enabling the use of those strains for specialized medical or biotechnological applications. This line of reasoning has been used by researchers to explain the probiotic profile of the *L. plantarum* YW11 strain. Some of the researchers used a particular technique, while others went in a completely different direction (Shin et al., 2022). Recent studies on them have given us more information about the probiotic potential of recently discovered species like *Lactobacillus helveticus* (Alessandri et al., 2022). It is difficult to assert that we have a firm grasp on the subject given the genetic pathways used to metabolize a wide variety of carbohydrates in the gut microbiota of newborns and adults, as well as the organism’s genomic plasticity. Even though we now have a better understanding of how *L. plantarum* YW11 adapts to the human GI tract, it would be premature to say that we currently have a firm grasp on the topic. The adaptability of the creature’s genome is responsible for this special quality. Therefore, it is crucial that this research includes genomes with distinctive traits. The genetic foundations of *L. plantarum* YW11, which survives in the various ecological niches that make up the human gut microbiome, are being investigated using comparative genomic analysis (Alessandri et al., 2022; Asarina et al., 2022; Chaudhary et al., 2022; Hebert and Meglécz, 2022; McPherson et al., 2022; Valdez-Baez et al., 2022; Wang et al., 2022; Xiang and Li, 2022). When analyzing these genetic roots, this context is very important. It also contains four additional strains that were isolated from young people in Chile and demonstrated a broad range of adaptability to the host using an *in-silico* method. Chile provided the first mention of the appearance of these novel strains (Alessandri et al., 2022; McPherson et al., 2022; Wang et al., 2022).

To this end, we attempted to characterize the functional genes of *L. plantarum* YW11 and other genomes with reported probiotic effects, in addition to other biological traits that may relate to the distinct host health advantages of this strain. We also checked for things like hydrophobic cell walls, antibiotic resistance, and antagonistic potential, and we examined cell growth. In that study, *L. plantarum* was shown to be resistant to the antibiotics cefoxitin, erythromycin, and metronidazole; to have a high inhibition rate against pathogens (including Listeria monocytogenes, Clostridium difficile, Vibrio cholera, and others); and to have a high aerotolerance, which is an advantageous property for industrial processes (Alessandri et al., 2022; Asarina et al., 2022; McPherson et al., 2022; Valdez-Baez et al., 2022; Wang et al., 2022). The genome project’s findings may shed light on some of these traits and mechanisms, paving the way for future research. The purpose of this research was to conduct a comparative genome analysis of this strain with 10 previously sequenced whole genomes of the species, and by searching for genes associated with favorable features.

## 2. Materials and methods

### 2.1. Analysis of *Lactiplantibacillus plantarum* YW11 comparative genome

The whole of the *L. plantarum* YW11 genome was submitted to GenBank and assigned the accession number.^1^ The nucleotide FASTA format was utilized in order to retrieve all 10 of the entire genome sequences of *L. plantarum* that can be found in the NCBI GenBank database (Hebert and Meglécz, 2022). All genomes were annotated using Prokka v1.14.5. The *L. plantarum* YW11 genome, along with the other fully sequenced genomes of the species, was used to conduct a synteny analysis. Several whole-genome sequence alignments were performed with the help of the implemented version of Mauve (v2.4) (Chaudhary et al., 2022).

### 2.2. Antibiotic resistance genes prediction

The NCBI-AMRFinderPlus, CARD, ARG-ANNOT, Resfinder, and MEGARES 2.0 databases were searched using the ABRIcate v1.0.1 software (Xiang and Li, 2022) in order to locate antibiotic resistance genes for the purpose of validating the accuracy of antibiotic resistance gene prediction (last update of databases: September 2022).

### 2.3. Taxonomy, phylogenomics, and evolutionary analysis

Calculations were made to determine the average levels of nucleotide similarity (ANI) between the 10 genomes of *L. plantarum* and the outgroup species (Alkalay-Oren et al., 2022). The phylogenetic tree was constructed by applying the Codon Tree Test method developed by the Pathosystems Resource Integration Center (PATRIC) (^2^viewed on 28 September 2022) to many genes, each of which only had a single copy of the gene (Spergser et al., 2022). This allowed for the phylogenomic tree to be accurate and reliable. The RaxML program utilized a total of 100 repetitions in order to calculate the support values (Batarseh et al., 2022).

### 2.4. Pangenome analysis

Data for 10 genomes retrieved from the NCBI RefSeq database were analyzed by panX to do the computation for the pangenome size (Ding et al., 2018). The analysis with the default settings and an identity cut-off of 99% was run. This was done while taking into consideration an abnormally high average GC content, which was equal to two times the standard deviation. The Cluster of Orthologous Genes (COG) designations were used to carry out the functional analysis and to explore the evolutionary relationships between gene clusters, and to identify potentially related gene clusters (Kamau et al., 2020). The analysis was used to illustrate the number of distinct genes possessed by each *L. plantarum* strain, and to analyze the biosynthetic pathways of gene clusters, and to identify potential new pathways.

### 2.5. Identifying genes related to probiotic features

The research that has been conducted on the genera *L. plantarum* and *Lactobacillus* has resulted in the discovery of genes that are involved in the mechanisms of adhesion, resistance to stress conditions (acid, bile salts, heat, and osmotic), the repair and protection of DNA and proteins, and the production of vitamins. These genes are also responsible to produce vitamins. Using a piece of software known as the Basic Local Alignment Search Tool (BLAST),^3^ we were able to match the protein sequences of these genes with the genome that we are now researching (Gaina et al., 2022). The alignment has to achieve at least 70% identity and a cutoff of 1E5 to be successful.

## 3. Results

### 3.1. Antibiotic resistance genes prediction

**Figure 1:**
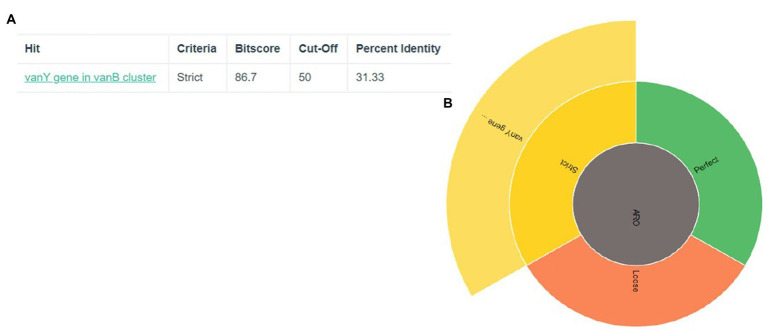
Prediction of antibiotic resistance genes **(A)** The criteria, cut-off and percent identity **(B)** the vanY gene show in strict area of ARO.

The discovery of genes that confer resistance to antibiotics led to the identification of two genes in total: vanY and vanB in Figure 1. The significance of the coverage percentage for each and every hit was more than 91.42 percent (Table 1).

**Table 1:** Resistance gene identification.

RGI criteria	ARO term	SNP	Detection criteria	AMR gene family	Drug class	Resistance mechanism	% identity of matching region	% length of reference sequence
Strict	VanY gene in vanB cluster		Protein homology model	vanY, glycopeptide resistance gene cluster	Glycopeptide antibiotic	Antibiotic target alteration	31.33	91.42

### 3.2. 3.2. Multiple whole genome sequence alignments

*L. plantarum* YW11 had a circular chromosome that was 2.99 Mbp in size and had 44.5% GC in its genome when it was completely sequenced. The genome assembly started off with a total of six contigs and a N50 value of 2,991,907. However, after the gaps in the sequence were filled in, it was able to retrieve the whole genome in a single contig. During the annotation procedure, a total of 2,832 genes, 68 transfer RNAs, 16 ribosomal RNAs, and 1986 CDS were found. The CDS represented 907 putative proteins. Concerning the origin of the data, most samples were collected from the feces of children, while just a few were taken from the feces of adults, vagina, the environment, and human breast milk. *L. plantarum* YW11 demonstrated collinearity of the gene blocks with most of the other genomes that were assessed while the conservation of the structure of the genome was being evaluated. In this regard, additional *L. plantarum* strains exhibited both a major and a small inversion in the genome’s core region, respectively.

### 3.3. Phylogenomic analysis

**Figure 2:**
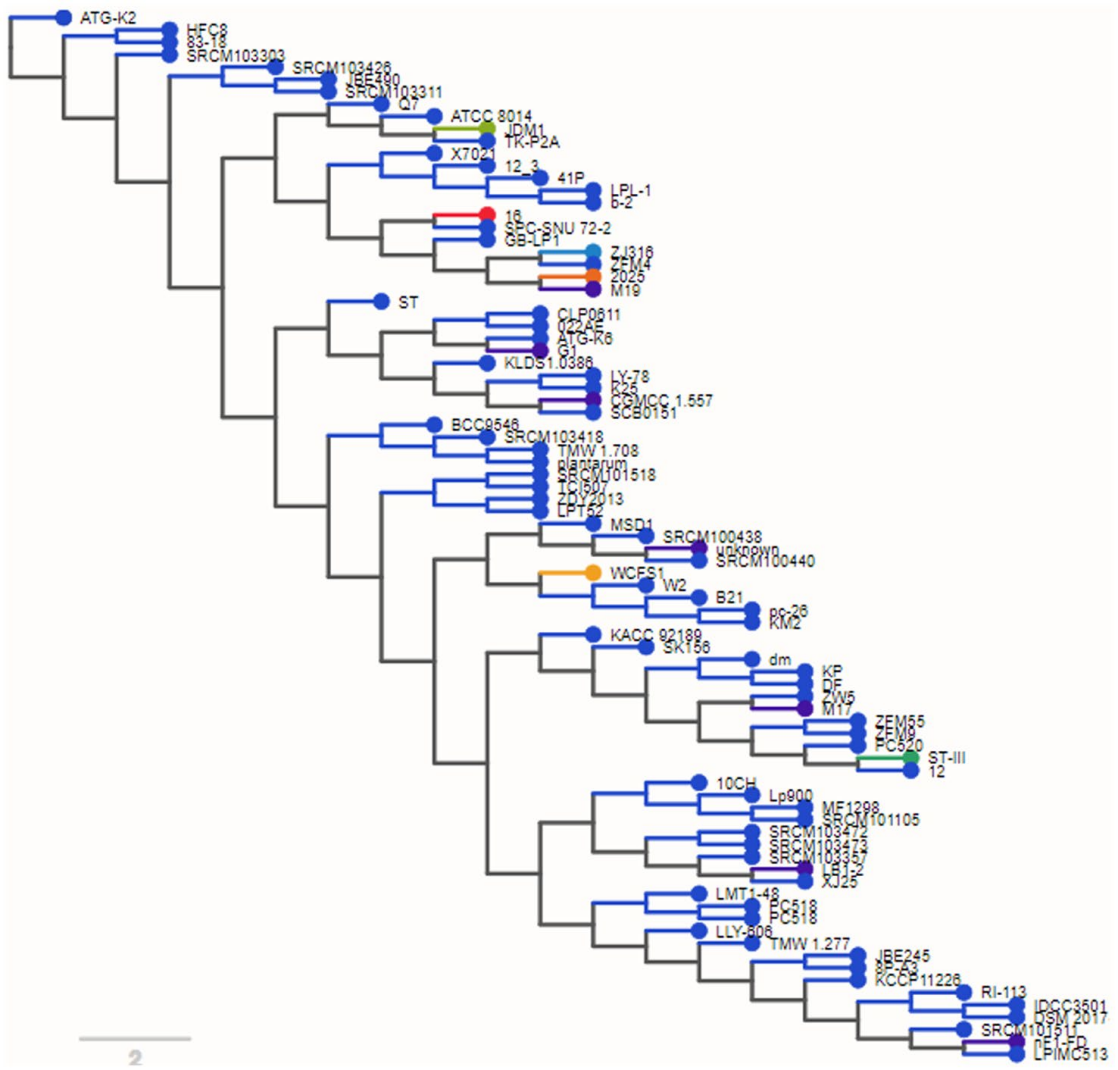
Phylogenetic tree shows genomes into clusters according to their taxonomic analysis.

The phylogenetic tree organized the genomes into clusters according to their prior taxonomic structure. This was done to represent the divergence that occurred among the branches of the subspecies that all descended from the same ancestor as shows in Figure 2. We found, as was to be predicted, that most genomes were located in a manner that allowed for uniform segregation into subspecies plantarum taxonomic categories.

### 3.4. Average nucleotide identity

**Figure 3:**
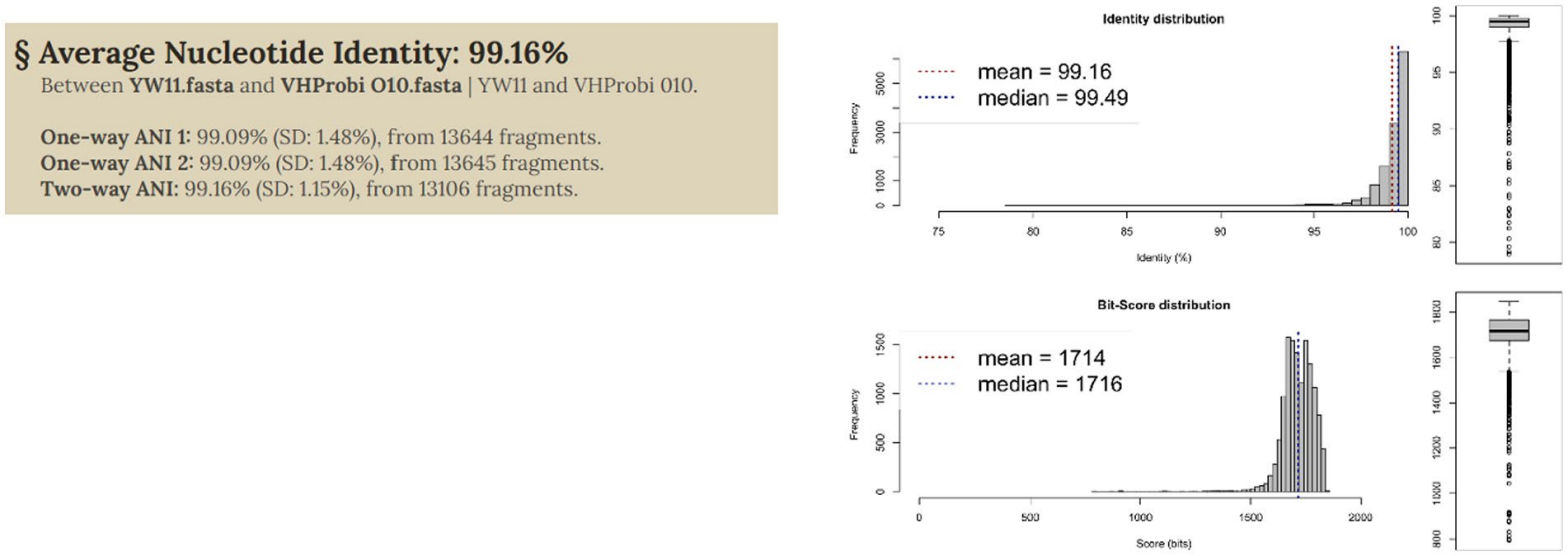
ANI analysis of genome to shows genomic relationship.

In order to assess the genomic link between the several *L. plantarum* genomes, an average nucleotide identity (ANI) analysis was carried out on each of the genomes that were selected for this research (both from public databases and the novel strains). This was done in order to define the genomic relationship among the *L. plantarum* genomes. The genomes were found to be substantially grouped into an ANI structure, with values reaching more than 0.991 as shows in Figure 3. It is interesting to note that some genomes came out with an ANI range lower than 0.994. Other strains of *L. plantarum*, for example, were isolated from calf feces and had the lowest ANI value. This elucidates the genetic difference that exists between strains that occupy the animal gut microbiome and those that inhabit the human gut microbiome. When it came to *L. plantarum* YW11, the ANI value was close to 0.995.

Figure 3 illustrates the taxonomic characterization achieved by doing similarity comparisons based on ANI values that were computed for each of the 10 strains of *L. plantarum*. In every single one of the comparisons that *L. plantarum* YW11 underwent with other strains of *L. plantarum*, the ANI values ranged between 0.94 and 0.96 when grouped with these strains, showing its high level of nucleotide similarity with this species.

### 3.5. Probiotic genes identification

**Figure 4:**
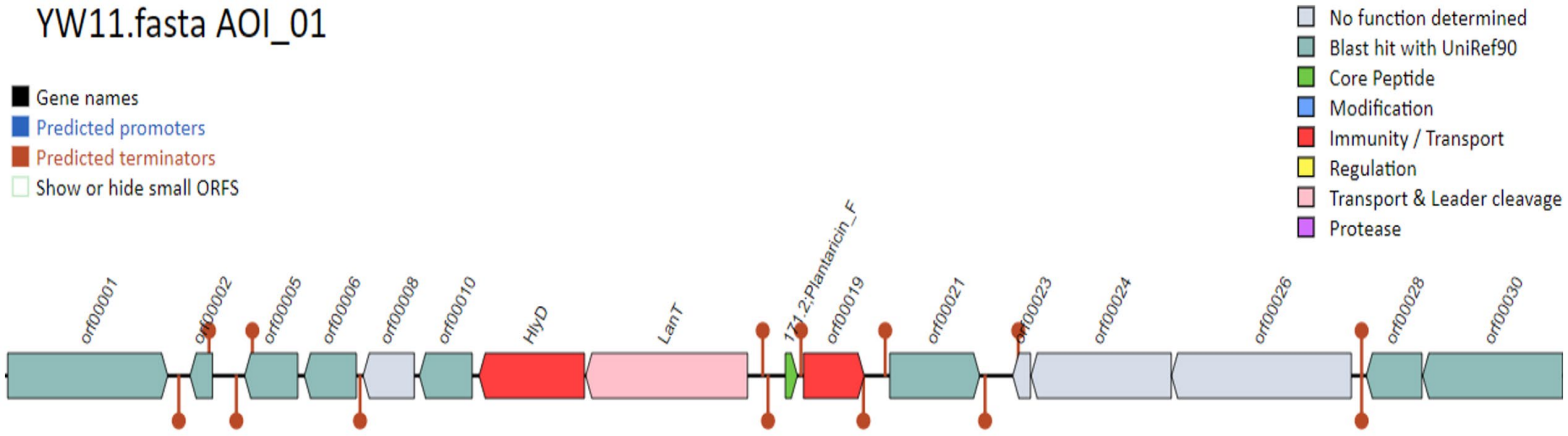
The gene names and their functions.

According to the display run summary and file information in Tables 2,3, the investigation uncovered a total of 16 condensed genes that were associated with adhesion. Among these genes were sequences that codified for sortases. The condensed genes include Immunity protein membrane-bound protease CAAX family, Glycerol uptake facilitator protein 3 OS = *Lactobacillus plantarum*, DNA helicase IV, and Alpha-glycerophosphate oxidase as display in Figure 4. Two sequences were codified for Accessory factor for ABC-transporter PlnH and Bacteriocin ABC-transporter, ATP-binding and permease protein PlnG shown in Table 4.

**Table 2:** Run summary for the analysis.

Run summary	
Number of Files analyzed	2
Number of DNA fragments analyzed	1
Total bases in all DNA	2,991,907
Number of Areas of Interest (AOI’s)	1

**Table 3:** The file name, class, and start and end.

AOI	Start	End	Class	Filename
NZ_CP0350311.0.AOI_01	261,713	2,652,058	171.2; Plantaricin_F	undefined

**Table 4:** The name of probiotic genes, function, and Motifs.

Name	Function	Motifs
orf00001	DNA helicase IV OS=Bacillus subtilis (strain 168) OX = 224,308 = held PE = 1 SV = 1	
orf00002	PlnY	
orf00005	PlnS	
orf00006	PlnS	
orf00008		
orf00010	PlnS	
HlyD	Accessory factor for ABC-transporter PlnH	PF13437
LanT	Bacteriocin ABC-transporter, ATP-binding and permease protein PlnG	PF00005; PF03412
171.2; Plantaricin_F	ggmotif; Lactococcin; Bacteriocin_llc; 171.2; Plantaricin	PF04369; PF10439
orf00019	P71468_LACPL Plnl, (Immunity protein membrane-bound protease CAAX family)	
orf00021	Transposase for insertion sequence element IS905 OS = Lactococcus lactis subsp. Lactis (strain IL1403) OX = 272,623 GN = tra905 PE = 3 SV = 1	
orf00023		
orf00024		
orf00026		
orf00028	Glycerol uptake facilitator protein 3 OS = *Lactobacillus plantarum* (strain ATCC BAA-793/ NCIB 8826 / WCFS1) OX = 220668 GN = glpF3 PE = 3 SV = 1	
orf00030	Alpha-glycerophosphate oxidase OS = Enterococcus Casseliflavus OX = 37,734 GN = glpO PE = 1 SV = 1	

### 3.6. Binary pan genome

**Figure 5:**
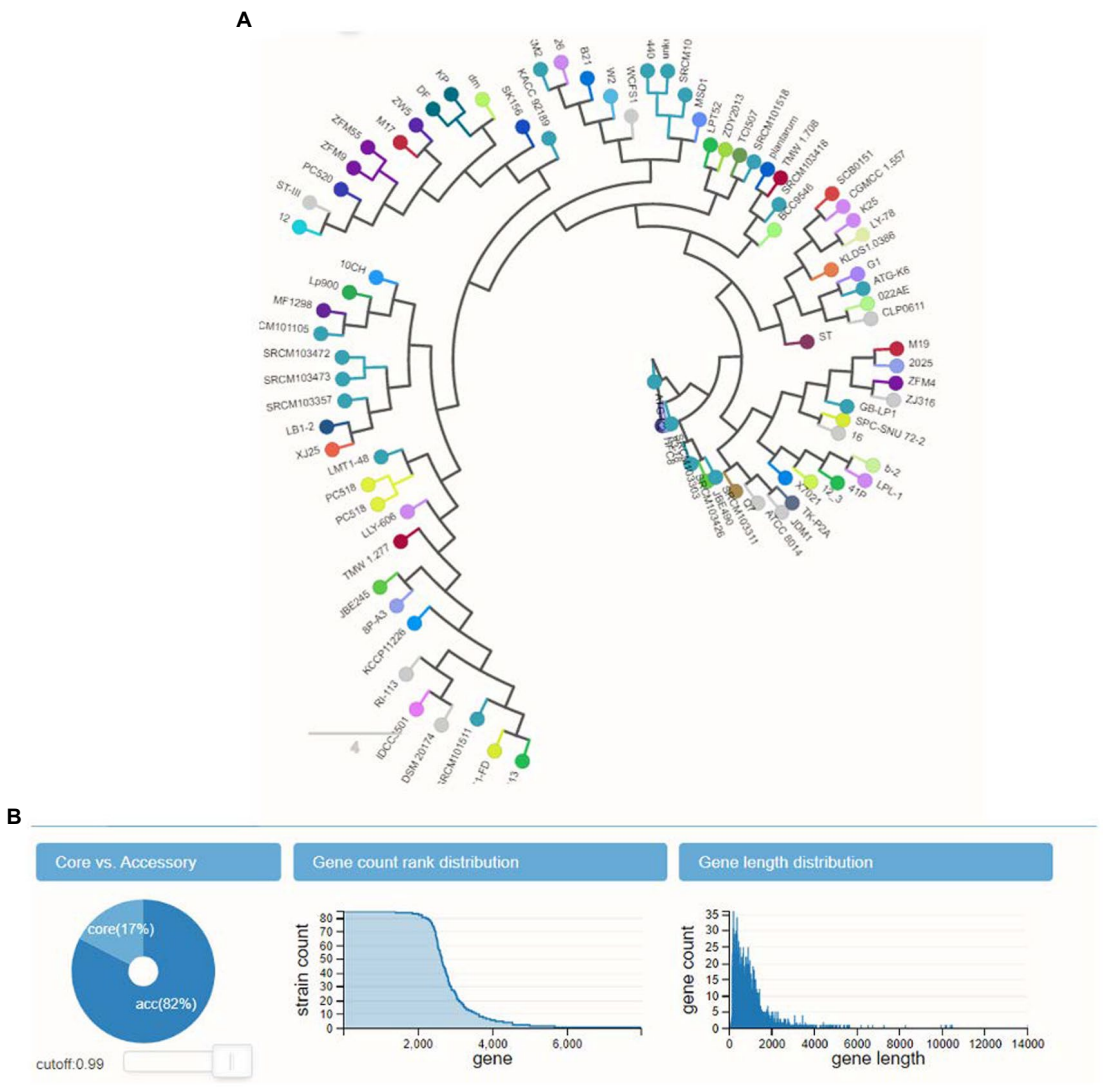
The panX outcomes for the pangenome analysis and exploration. **(A)** *L. plantarum* strains were shown to be effective probiotics and revealed a connection to other clades **(B)** the gene counts distribution and length distribution analysis.

The calculation of the size of the pangenome found a total of 4,477 genes, based on how they were distributed throughout the 10 genomes. According to the ANI analysis, the fact that *L. plantarum* YW11 formed a well-supported clade with *L. plantarum* YW11 81 in the phylogenomic tree that was constructed using single-copy genes suggests that this strain is closely related as shows in Figure 5B. While performing experiment, L. plantarum strains were shown to be effective probiotics and revealed a connection to other clades as display in Figure 5A.

## 4. Discussion

Comparative genomics studies on various strains of *L. plantarum* may provide information on how different taxonomic groups adapt to their habitat and which of their traits are required for such adaptations. These modifications might be related to the host or to the geological and geographical environment in which they dwell. The taxa of *L. plantarum* exhibit greater genomic variety than previously believed, according to earlier findings from pangenome research (Kamau et al., 2020; Gaina et al., 2022; Li et al., 2022). A closed pangenome is regarded as a finalized pangenome in which the number of genomes does not change even if new genomes are added to it, as opposed to an open pangenome, that expands every time a new genome is added. It has been proposed that whether the pangenome is open or closed is closely tied to the mode of life of the bacterial species being studied (Surve et al., 2022). Given this perspective, animals with an open pangenome are which live in various habitats and have a variety of genetic exchange pathways. Salmonellae, Escherichia coli, *Helicobacter pylori, Streptococci,* and *Meningococci* pangenomes are a few examples. As a result, they have a restricted selection of genes available to them. Examples of closed pangenomes are Mycobacterium TB, Bacillus anthracis, and Chlamydia trachomatis (Gaina et al., 2022; Li et al., 2022; Liu et al., 2022; Surve et al., 2022; Syrokou et al., 2022).

The gastrointestinal tract includes the oral cavity, large intestine, stomach, and small intestine of the human are some of the locations where *L. plantarum* YW11 may be found. It stands out among gut microbes because it is a major component of the gut microbiota in newborn humans and is frequently found in the gut microbiota of adults (Teame et al., 2020). Humans are the only species with this characteristic (Kim et al., 2022). Previous research indicates that by examining the core genome, which is a genetically conserved section, we may be able to identify subspecies-specific adaptations. The COGs discovered in each strain isolated from Chileans in this study were found to be among the higher percentages allocated to the functional category “carbohydrate transport and metabolism (G)” in the shell gene set (Li et al., 2022). The metabolism of carbohydrates falls under this category. To acquire nutrients and subsequently carve out an ecological niche for themselves, these functions are crucial in controlling the contact with the host and the environment (Carpi et al., 2022; Yin et al., 2022; Aziz et al., 2023).

It’s remarkable how different conclusions can be drawn from the phylogenetic analysis of *L. plantarum* YW11. The *L. plantarum* YW11 strain was classified as a subspecies of *lactobacillus* in both the phylogenetic tree we built and the original annotation. *L. plantarum* YW11, which was isolated from an infant’s gut microbiota, thrived in neutral HMOs such as LNT and LNnT. Previous studies suggest *L. plantarum* YW11 may be a niche adaptation rather than a horizontal gene transfer (Syrokou et al., 2022). *L. plantarum* YW11 also grouped further away from Chilean isolated strains with an ANI value <0.98. Its genome is closer to other *L. plantarum* strain, which was isolated from a calf’s stomach microbiota. Albert et al. study’s grouped the *L. plantarum* YW11 genome like the infantis subspecies. Although most *L. plantarum* genomes belong to the subspecies *lactobacillus*, some strains, such as YW11, may have had a unique genomic architecture to adapt to their ecological niches (Spergser et al., 2022).

## 5. Conclusion

In our recently published study we demonstrated that the *L. plantarum* YW11 genome we found exopolysaccharides including terpenes, T3PKS and RiPP like regions. On further investigations of this genome with other species, e.g, *enterococcus*, *bacillus cereus* and *halo bacillus* we noticed that *L. plantarum* YW11 genome has two bacteriocins Streptin and Ruminococcin-A, were further analyzed for their probiotic role *via* docking with virulent proteins of pathogenic bacterial species which confirmed that both bacteriocins are potent inhibitors of the target bacterial pathogens and help the human host elicit a strong immune response against pathogenic bacteria. In this study we found out that a carbohydrate enzyme in the *L. plantarum* YW11 genome. Similar enzymes were discovered in *L. plantarum* strains during previous studies. The previous research demonstrated that particular strains of *L. plantarum* may selectively constrain the development of the baby’s gut microbiota’s carbohydrate-mediated symbiosis. The conclusions that were reached from the study reflected these findings. *L. plantarum’s* metabolic abilities are critical for trophic interactions with other commensal bacterial populations, promoting a mutualistic environment in their host, allowing cross-feeding connections between microorganisms, and maintaining appropriate gut microbiome growth. Cross-feeding interactions are those in which one microbe consumes the nutrients from another microbe. This genome *L. plantarum* YW11 is of very great interest and can be helpful for food safety, food fermentation and food starter cultures. Moreover, its probiotic capabilities cannot be ignored and several *in-vitro* and *in-vivo* activities can be performed on it.

## Data availability statement

The datasets presented in this study can be found in online repositories. The names of the repository/repositories and accession number(s) can be found in the article/supplementary material.

## Author contributions

TA, MN, KJ, MS, and YZ: conceptualization. AA, MN, KJ, MS, and YZ: methodology and investigation. MA: software. AA: validation. TA: formal analysis and data curation. YZ, MA, and AA: resources. TA and MN: writing—original draft preparation and writing—review and editing. KJ, AS, AA, and MS: visualization. YZ: supervision and funding acquisition. AA and MA: project administration. All authors contributed to the article and approved the submitted version.

## Funding

This research work was financially supported by National Natural Science Foundation of China (Grant no. 31871823) and National Key Research and Development Program of China (2017YFE0131800).

## Conflict of interest

The authors declare that the research was conducted in the absence of any commercial or financial relationships that could be construed as a potential conflict of interest.

## Publisher’s note

All claims expressed in this article are solely those of the authors and do not necessarily represent those of their affiliated organizations, or those of the publisher, the editors and the reviewers. Any product that may be evaluated in this article, or claim that may be made by its manufacturer, is not guaranteed or endorsed by the publisher.

## Footnotes

^1^ https://www.ncbi.nlm.nih.gov/assembly/GCF_004028295.1

^2^ http://www.patricbrc.org

^3^ https://blast.ncbi.nlm.nih.gov/Blast.cgi
